# Transcription factor Yin Yang 2 is a novel regulator of the p53/p21 axis

**DOI:** 10.18632/oncotarget.18005

**Published:** 2017-05-19

**Authors:** Vivi Kasim, Yu-Dan Xie, Hui-Min Wang, Can Huang, Xue-Song Yan, Wei-Qi Nian, Xiao-Dong Zheng, Makoto Miyagishi, Shou-Rong Wu

**Affiliations:** ^1^ The Key Laboratory of Biorheological Science and Technology, Ministry of Education, College of Bioengineering, Chongqing University, Chongqing, China; ^2^ The 111 Project Laboratory of Biomechanics and Tissue Repair, College of Bioengineering, Chongqing University, Chongqing, China; ^3^ Chongqing Cancer Institute, Chongqing, China; ^4^ Molecular Composite Medicine Research Group, Biomedical Research Institute, National Institute of Advanced Industrial Science and Technology (AIST), Tsukuba, Japan

**Keywords:** cell cycle, tumor suppressor, p53, p21, Yin Yang 2 (YY2)

## Abstract

Yin Yang 2 (YY2) is a multifunctional zinc-finger transcription factor that belongs to YY family. Unlike the well-characterized YY1, our understanding regarding the biological functions of YY2 is still very limited. Here we found for the first time that in contrast to YY1, which had been reported to be oncogenic, the expression level of YY2 in tumor cells and/or tissues was downregulated compared with its expression level in the normal ones. We also demonstrated that YY2 exerts biological function contrary to YY1 in cell proliferation. We elucidated that YY2 positively enhances p21 expression, and concomitantly, its silencing promotes cells to enter G2/M phase and enhances cell proliferation. Furthermore, we found that YY2 regulation on p21 occurs p53-dependently. Finally, we identified a novel YY2 binding site in the promoter region of tumor suppressor p53. We found that YY2 binds to the p53 promoter and activates its transcriptional activity, and subsequently, regulates cell cycle progression via p53/p21 axis. Taken together, our study not only identifies YY2 as a novel tumor suppressor gene that plays a pivotal role in cell cycle regulation, but also provides new insights regarding the regulatory mechanism of the conventional p53/p21 axis.

## INTRODUCTION

Yin Yang (YY) protein family is a zinc finger protein family that contains four highly conserved C2H2 zinc finger domains. The firstly identified member of the family, Yin Yang 1 (YY1), could activate or repress transcription depending upon the cellular content in which it binds to recruit cofactors [1, 2]. Furthermore, recent reports showed that it is also involved in post-transcriptional gene regulation [3, 4]. YY1 had been reported to play crucial roles in various biological processes, including embryonic development, cell cycle, cell proliferation and apoptosis [5–9]. YY1 had also been reported as an oncogene, as its overexpression is observed in various cancers and correlated with poor prognosis, most plausibly through its role in promoting proliferation and colonialization of tumor cells, as well as tumor angiogenesis [3, 4, 7, 10–12].

A more recent study reported the existence of YY2, another member of YY family with 56.2% identity to YY1 in their overall amino acid sequences and 86.4% high identity in the highly conserved carbon terminal DNA-binding zinc-finger protein domains [13, 14]. YY2 is an intronless, retroposed copy of the YY1 mRNA that had been inserted into the intronic segment between exon 5 and 6 of membrane bound transcription factor peptidase, site 2 (Mbtps2) gene. Unlike YY1 which is evolutionarily well conserved throughout all vertebrate lineages, it is most likely that YY2 was retroposed recently after the divergence of placental mammals from other vertebrates, and thus, YY2 is unique to placental mammals [15]. Furthermore, previous studies have demonstrated that YY1 and YY2 showed different expression patterns, for example during brain development and in ovary follicles, sperm cells, granular layers of cerebellum, as well as in neuronal and glial cells [15, 16]. These facts indicate that YY1 and YY2 might be different in both transcriptional regulations and biological functions. However, despite that YY1 has been known to be involved in various biological pathways, and that YY1 and YY2 share high structural similarity, our knowledge regarding the biological functions of YY2 is still very limited.

In this study, we explored the roles of YY2 in tumorigenesis, especially in the regulation of cell cycle and cell proliferation. We revealed that in contrast to YY1 which could induce tumorigenesis, YY2 is a novel tumor suppressor which binds to the promoter region of tumor suppressor p53, promotes its transcriptional activity, activates the p53/p21 axis, and subsequently, suppresses cell cycle progression and cell proliferation.

## RESULTS

### YY2 expression is downregulated in tumor tissues

**Figure 1:**
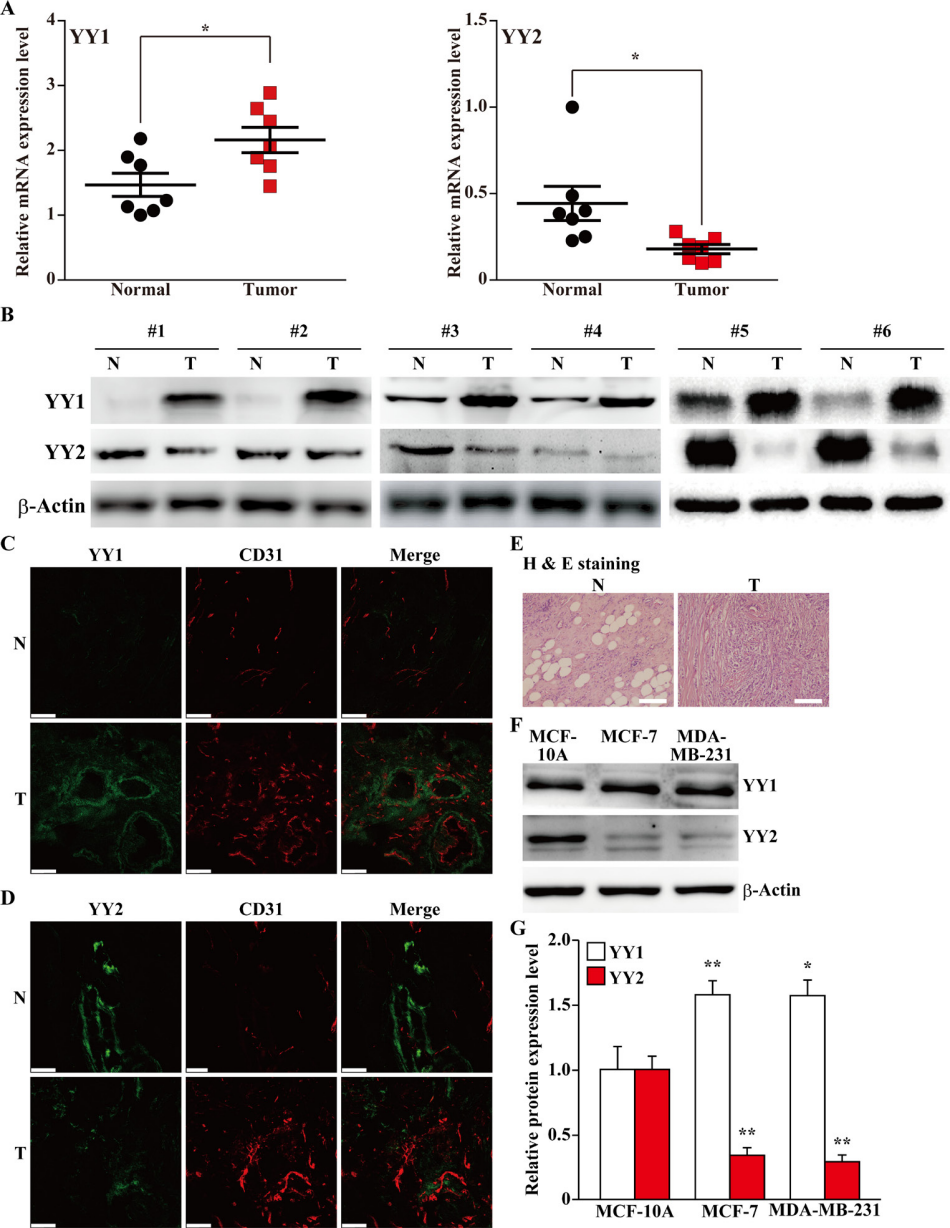
Distinct expression patterns of YY family in tumor tissues (**A**, **B**) The expression levels of YY1 and YY2 in clinical human breast carcinoma tissues: (A) mRNA expression levels of YY1 and YY2 were examined using quantitative RT-PCR (qPCR, *n* = 7); (B) protein expression levels were examined using western blotting. (**C**, **D**) Representative immunohistochemical staining images of the clinical human breast carcinoma tissues for YY1 (C) or YY2 (D) and CD31. YY1 and YY2 were shown in green, while CD31 was shown in red. (**E**) Hematoxylin and eosin staining of clinical human breast carcinoma tissues and normal adjacent tissues. (**F**, **G**) Western blotting images (F) and their quantification results (G) showing the protein expression levels of YY1 and YY2 in normal mammary epithelial cell line (MCF-10A), and mammary tumor cell lines (MCF-7 and MDA-MB-231). The protein expression levels were shown as relative to those in MCF-10A. β-Actin was used for normalization in qPCR, and as a loading control in western blotting. Quantitative data were shown as mean ± S.D. Scale bars: 200 μm; N: normal adjacent tissue; T: tumor tissue; *P < 0.05; **P < 0.01.

To elucidate the role of YY2 in tumorigenesis, we first compared the expression levels of YY1 and YY2 in human clinical breast carcinoma tissues and the corresponding normal adjacent tissues. As shown in Figure 1A, contrary to YY1, which showed a significant increase in tumor tissues (left panel), the mRNA expression level of YY2 was robustly decreased (right panel). Consistently, western blotting results demonstrated that the protein expression level of YY2 showed a pattern completely different from that of YY1, as it was lower in tumor tissues than in normal adjacent tissues (Figure 1B). Immunohistochemical staining results further confirmed these tendencies: compared with normal adjacent tissues, the number of YY1 positive cells was significantly higher in tumor tissues (Figure 1C), while the number of YY2 positive cells robustly decreased (Figure 1D). The histological characteristic of the clinical samples used was confirmed using hematoxylin and eosin staining (Figure 1E).

Next, to further confirm the different expression profiles of YY1 and YY2 in normal and tumor cells, we compared the expression levels of YY1 and YY2 in normal mammary epithelial cell line (MCF-10A) and breast cancer cell lines (MCF-7 and MDA-MB-231). We observed an increase of YY1 protein expression level in MDA-MB-231 and MCF-7 cells compared with MCF-10A cells; while YY2 expression level in breast cancer cell lines was robustly decreased (Figure 1F, 1G). Together, these data consistently showed that YY2 expression was lower in tumor cells and tissues, and thus might be involved in tumorigenesis in the manner totally different from that of YY1.

### YY2 negatively regulates cell cycle and proliferation by promoting p21 transcription level

To further analyze the biological functions of YY2 in tumorigenesis, we constructed two shRNA expression vectors against YY1 and two shRNA expression vectors against YY2, and examined their silencing effects in MCF-7 cells. Transfection of shRNA expression vectors efficiently reduced YY1 and YY2 expression levels (Supplementary Figure 1A–1D).

**Figure 2:**
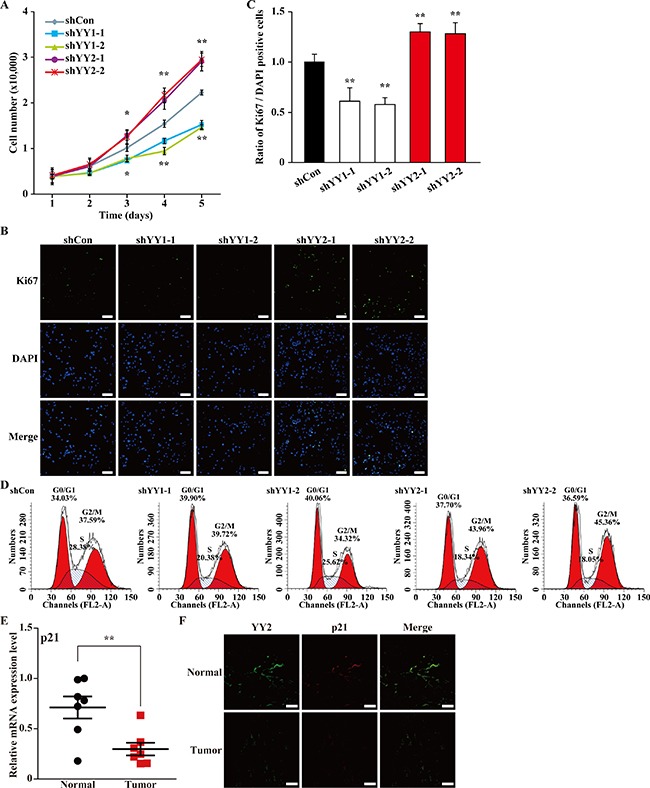
YY1 and YY2 exert opposite roles in cell cycle regulation (**A**) Total number of MCF-7 cells transfected with shYY1 or shYY2 and selected with puromycin at indicated time points. Data were expressed as mean ± S.D. (*n* = 3). (**B**, **C**) The proliferation of MCF-7 cells transfected with shYY1 or shYY2 was examined by using Ki67 staining: (B) representative images of Ki67 staining; (C) quantification of the ratio of Ki67 positive cells to DAPI positive cells. Scale bars: 100 μm. Quantification data were shown as relative to that of the cells transfected with shCon, and expressed as mean ± S.D. (*n* = 6). DAPI was used to stain nuclei. (**D**) Representative results of the percentage of MCF-7 cells transfected with shYY1 or shYY2 in each cell cycle phase examined by using propidium iodide staining and flow cytometry. (**E**) The mRNA expression level of p21 in clinical human breast carcinoma tissues was examined by using quantitative RT-PCR (*n* = 7). β-Actin was used for normalization. (**F**) The expression levels of YY2 and p21 protein in clinical human breast carcinoma tissues were examined using immunohistochemistry. Scale bars: 50 μm; *P < 0.05; **P < 0.01.

We next analyzed the effects of YY1- and YY2-silencing on cell proliferation. We first examined the numbers of YY1- and YY2-silenced cells at indicated time points. Consistent with our previous study, YY1-silencing resulted in a significant reduction of the total cell number [4]; however, interestingly, YY2-silencing resulted in a completely different effect, as it grossly increased the total cell number (Figure 2A). Accordingly, we hypothesized that YY2-silencing might alter cell proliferation potential in a manner different from that of YY1.

To further examine this possibility, we performed Ki67 staining. As shown in Figure 2B, compared to shCon, shYY2 transfection induced a robust increase of the number of Ki67 positive cells, while shYY1 transfection significantly decreased it. Consistently, quantification results showed that in contrast to YY1-silenced cells, the ratio of Ki67 positive cells to total cell number was conspicuously higher in YY2-silenced cells (Figure 2C).

Next, we analyzed the effect of YY2-silencing on the cell cycle progression by performing flow cytometry using propidium iodide staining. As shown in Figure 2D, compared to cells transfected with shCon (G0/G1 phase: 34.03%, S phase: 28.38%), in cells transfected with shYY1-1 and shYY1-2, the percentage of the G0/G1 phase cell population increased to 39.90% and 40.06%; while S phase cell population decreased to 20.38% and 25.62%, respectively. In contrast, YY2-silencing showed a totally different effect on cell cycle regulation, as YY2-silencing resulted in the decrease of S phase cell population (from 28.38% in control cells to 18.34% and 18.05%), and the increase of G2/M phase cell population (from 37.59% in control cells to 43.96% and 45.36%). These results indicated that YY2-silencing might promote S phase cells to enter G2/M phase. Together with the fact that YY2-silencing enhanced the number of Ki67 positive cells, our results showed for the first time that YY1 and YY2 play distinct roles on regulating cell cycle progression: YY2-silencing enhances cell cycle progression and eventually, cell proliferation, while YY1-silencing slowed down cell cycle progression by causing G0/G1 arrest.

p21 is a key factor that regulates cell cycle progression. Thus, we next examined its expression level in tumor specimens by using the tumor tissues and the adjacent normal tissues showed in Figure 1A, and found that the expression level of p21 robustly decreased in tumor tissues (Figure 2E). Immunohistochemistry staining results further confirmed that both YY2 and p21 expressions were lower in tumor tissues compared to their expressions in normal adjacent tissues (Figure 2F).

### YY2 positively regulates p21 transcriptional activity

**Figure 3:**
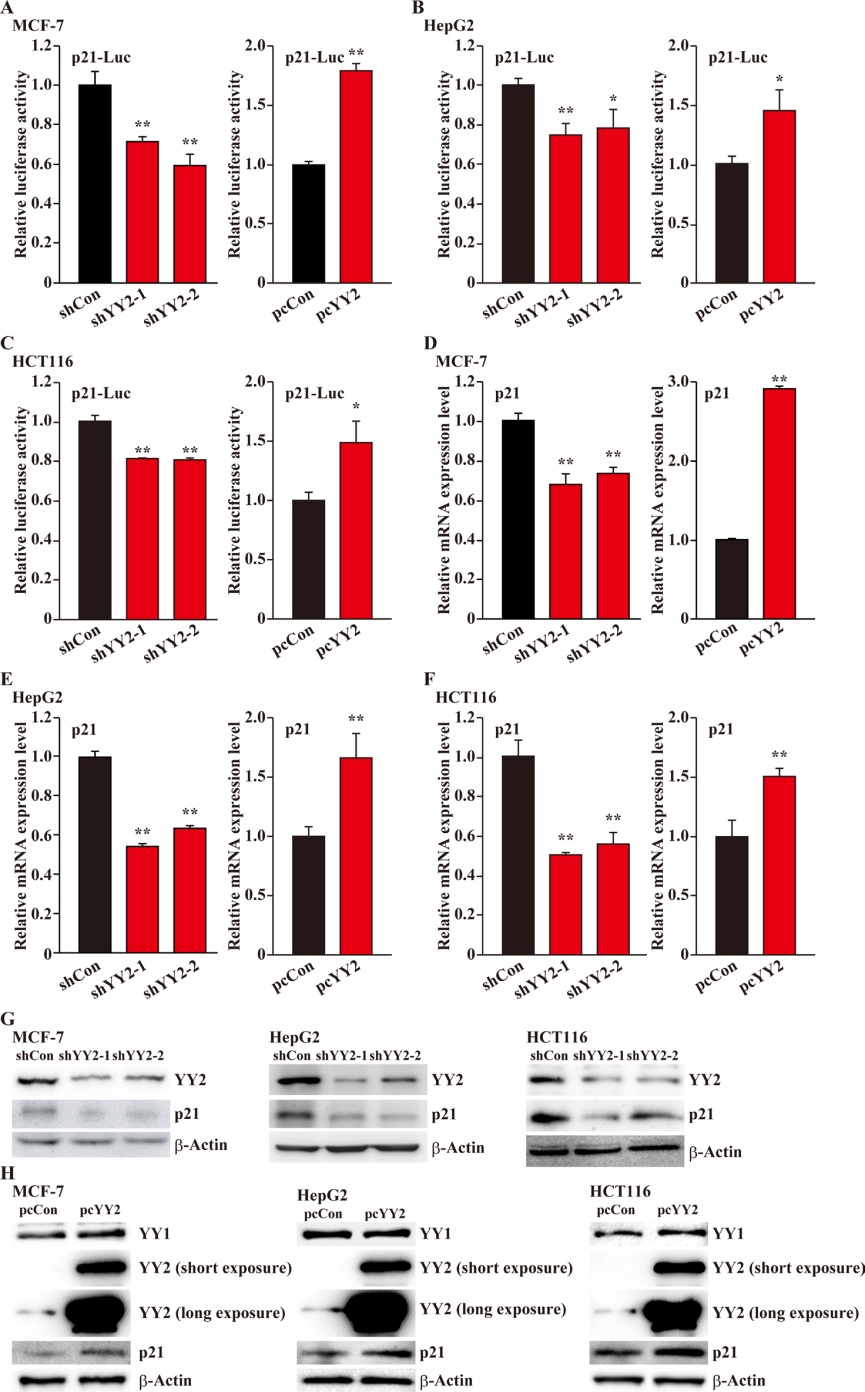
YY2 enhances p21 transcriptional activity (**A**–**C**) The effects of YY2 silencing (left panels) and overexpression (right panels) on p21 reporter activity in MCF-7 (A), HepG2 (B) and HCT116 (C) cells were examined by using dual luciferase reporter assay. Cells transfected with shCon or pcCon were used as controls. Luciferase activity was calculated as the ratio of the firefly and *Renilla* luciferase activities. (**D**–**F**) p21 mRNA expression level in YY2-silenced (left panels) and YY2-overexpressed (right panels) MCF-7 (D), HepG2 (E) and HCT116 (F) cells was analyzed by using quantitative RT-PCR. Cells transfected with shCon or pcCon were used as controls. β-Actin was used for normalization. (**G**, **H**) p21 protein expression level in YY2-silenced (G) and YY2-overexpressed (H) MCF-7, HepG2 and HCT116 cells was analyzed by using western blotting. For YY2 overexpression experiments, long exposure was performed to confirm the presence of endogenous YY2 bands. β-Actin was used as a loading control. Quantitative data were shown as relative to control and expressed as mean ± S.D. (*n* = 3). pcCon: pcDNA3.1; * P < 0.05, ** P < 0.01.

To further elucidate the molecular mechanism underlying the regulatory roles of YY2 on cell cycle progression, we next investigated the effect of YY2 expression level on the transcriptional activity of p21 in zvarious cancer cell lines. For this purpose, we constructed a p21-reporter vector by inserting the –95 to +2,555 region of p21 promoter into the upstream of firefly luciferase gene, and examined whether or not YY2 expression level affects the luciferase activity. It is noteworthy that the shRNAs targeting YY1 and YY2 used significantly suppressed the expression levels of YY1 and YY2 in all cell lines used (Supplementary Figure 1A–1F). As shown in Figure 3A, YY2-silencing in MCF-7 cells conspicuously suppressed the p21-reporter activity (left panel), while YY2 overexpression grossly induced it (right panel). These results conformed with the results of YY2-silencing and overexpression in HepG2 and HCT116 cells (Figure 3B and 3C, respectively). In contrast, YY1-silencing resulted in the induction of p21 reporter activity, while its overexpression decreased it (Supplementary Figure 2A–2C for MCF-7, HepG2 and HCT116, respectively). Concomitantly, YY2-silencing significantly reduced p21 mRNA expression level (left panels), and YY2 overexpression robustly increased it (right panels) (Figure 3D–3F for MCF-7, HepG2 and HCT116, respectively); while YY1 regulates p21 mRNA expression in a contrary manner (Supplementary Figure 2D–2F). The results of p21 protein expression levels further conformed these tendencies: in MCF-7, HepG2 and HCT116 cells, YY2-silencing resulted in the downregulation of p21 protein expression level, and YY2 overexpression significantly increased it (Figure 3G–3H); on the other hand, YY1-silencing robustly induced p21 protein expression level, while YY1 overexpression suppressed it (Supplementary Figure 2G–2H). Analysis of the expression levels of cell cycle related proteins cyclin A and cyclin dependent kinase 1 (CDK1), which are downstream targets of p21, further confirmed this regulatory effect: YY2-silencing robustly increased their expression levels (Supplementary Figure 3). Together, these results indicated that in tumor cells, YY2 might act as a p21 positive regulator, and thus negatively regulate cell cycle progression and cell proliferation.

### YY2 induces p21 expression in a p53 dependent manner

**Figure 4:**
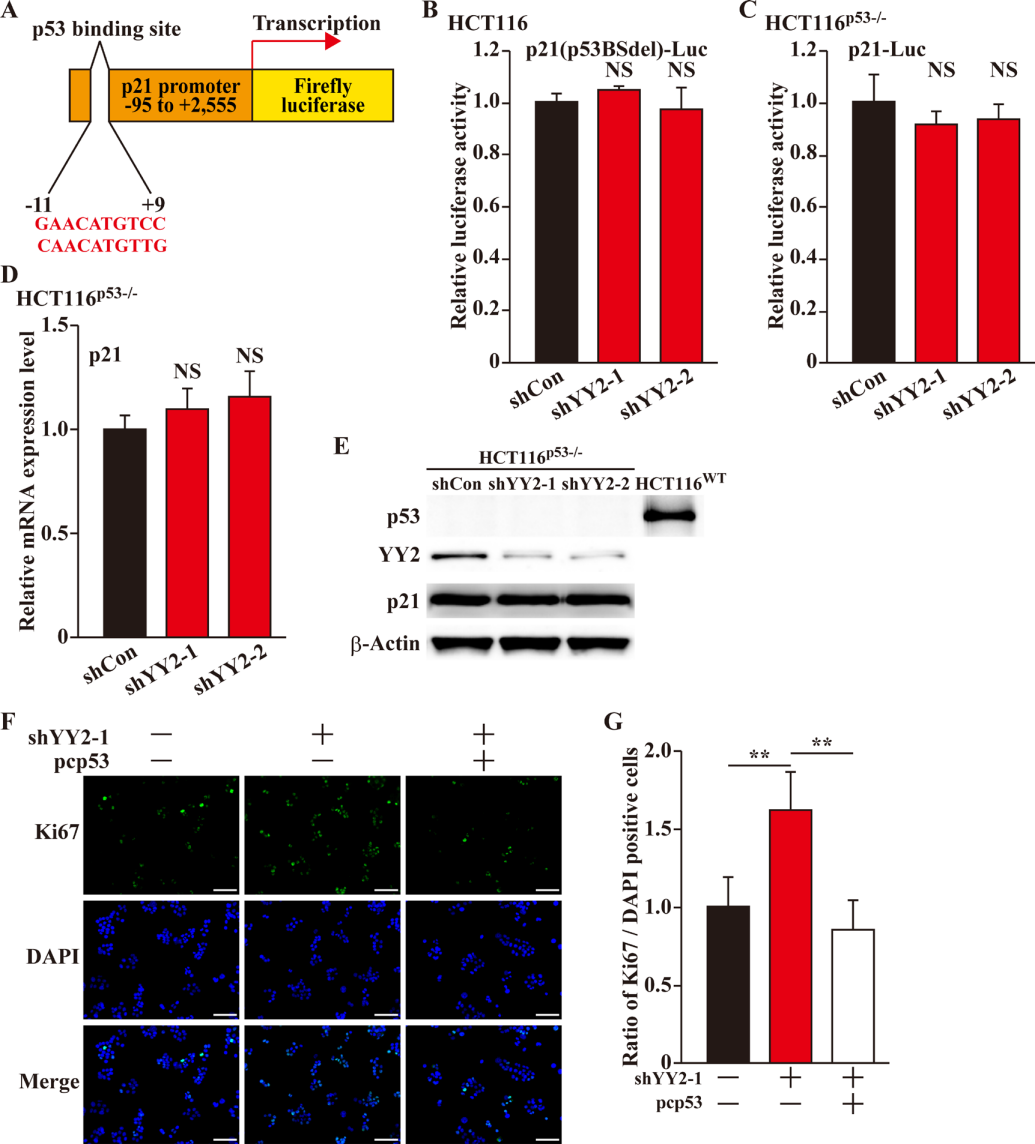
YY2 regulates p21 in a p53-dependent manner (**A**) Schematic diagram of p21-reporter vector lacking p53 binding site (p21(p53BSdel)-Luc). (**B**, **C**) Relative luciferase activity of p21(p53BSdel)-Luc in YY2-silenced HCT116 cells (B); and of wild-type p21-reporter vector (p21-Luc) in YY2-silenced HCT116^p53-/-^ cells (C). Cells transfected with shCon were used as control. Luciferase activities were calculated as the ratio of the firefly and *Renilla* luciferase activities. Data were shown as relative to control, and were expressed as mean ± S.D. (*n* = 3). (**D**, **E**) p21 mRNA (D) and protein (E) expression levels in YY2-silenced HCT116^p53-/-^ cells were examined by using quantitative RT-PCR and western blotting, respectively. Cells transfected with shCon were used as control. Data were shown as relative to control, and were expressed as mean ± S.D. (*n* = 3). (**F**, **G**) Proliferation of HCT116 cells transfected with shYY2 and pcp53 were examined by using Ki67 staining: (F) representative images of Ki67 staining; (G) quantification of the ratio of Ki67 positive cells to DAPI positive cells Scale bars: 200 μm. Data were shown as relative to control and expressed as mean ± S.D. (*n* = 6). DAPI was used to stain nuclei. Cells transfected with shCon and pcCon were used as control. β-Actin was used for normalization in qPCR, and as a loading control in western blotting. NS: not significant; **P < 0.01.

Tumor suppressor p53 had been known as a positive regulator of p21 transcriptional activity [17, 18]. Thus, we next investigated whether or not YY2 regulation on p21 transcription occurs through p53. First we constructed a p21-reporter lacking p53 binding site (p21(p53BSdel)-Luc, Figure 4A), and found that YY2-silencing could no longer affect the p21-reporter activity (Figure 4B). The same result was obtained when the wild-type p21-reporter was transfected into YY2-silenced p53-null HCT116 (HCT116^p53-/-^) cells (Figure 4C). Furthermore, YY2-silencing did not affect the mRNA and protein expression levels of p21 in HCT116 ^p53-/-^ cells (Figure 4D and 4E, respectively), indicating that YY2 regulates the expression level of p21 in a p53-dependent manner. Concomitantly, we found that while YY2-silencing could enhance cell proliferation, p53 overexpression could indeed repress it (Figure 4F, 4G). Together, these results indicated that YY2 regulation on p21, and subsequently, on the cell proliferation, occurs p53-dependently.

### YY2 binds to p53 promoter region and activates its transcriptional activity

**Figure 5:**
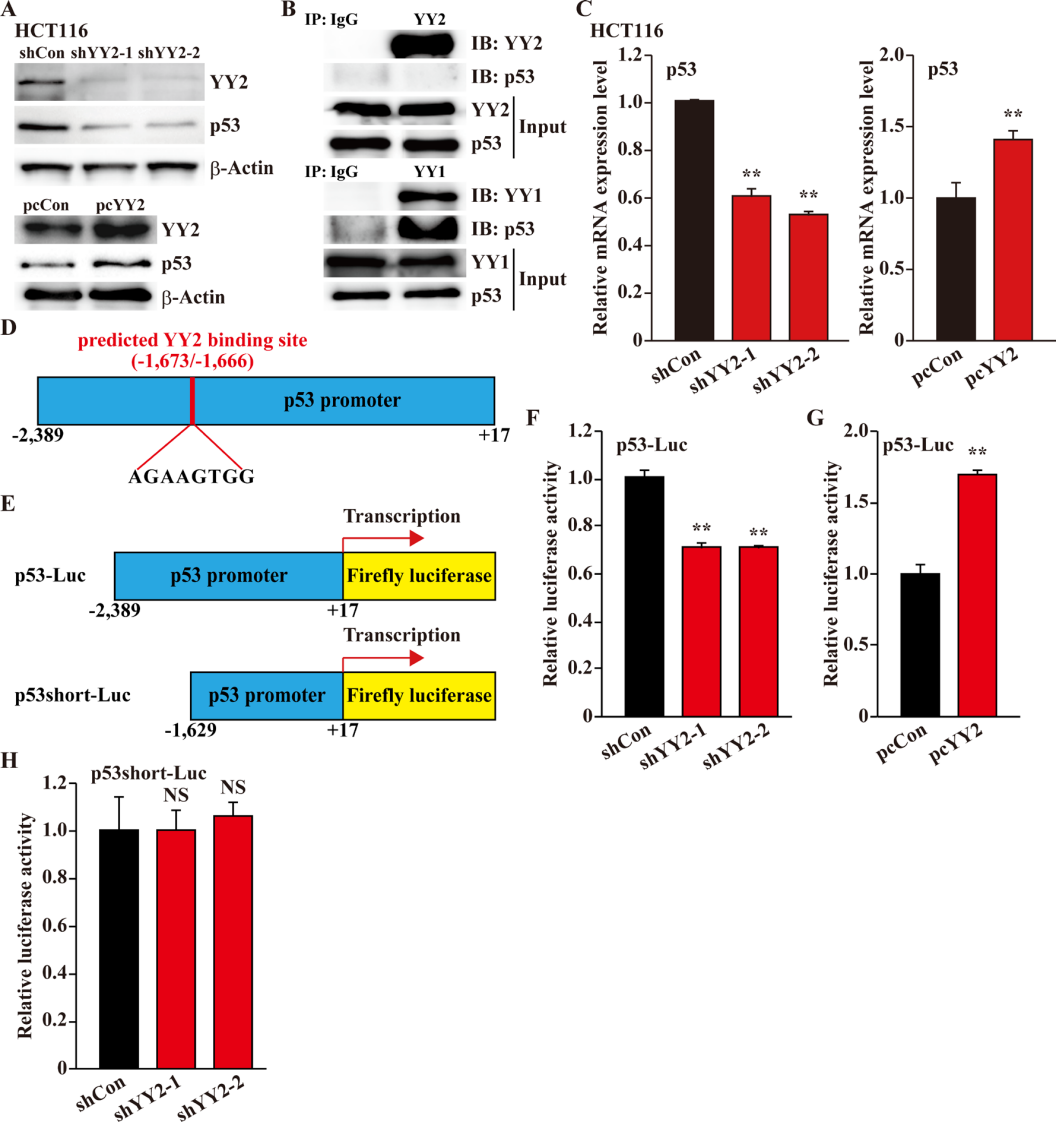
YY2 regulates p53 transcriptional activity (**A**) p53 protein expression level in YY2-silenced (top panels) or YY2-overexpressed (bottom panels) HCT116 cells was examined by using western blotting. (**B**) Physical interactions between YY2 (top panels) or YY1 (bottom panels) and p53 were examined using immunoprecipitation. The presence of p53 in the lysates of HCT116 cells immunoprecipitated with anti-YY1 or anti-YY2 antibodies was detected using immunoblotting against p53. (**C**) p53 mRNA expression level in YY2-silenced (left panel) or YY2-overexpressed (right panel) HCT116 cells was examined by using quantitative RT-PCR. Cells transfected with shCon or pcCon were used as controls. (**D**) Schematic diagram of predicted YY2 binding site on the promoter region of p53. (**E**) Schematic diagrams of wild-type p53-reporter vector (p53-Luc) and p53-reporter vector lacking the predicted YY2 binding site (p53short-Luc). (**F**, **G**) Relative luciferase activity of p53-Luc in YY2-silenced (F) or YY2-overexpressed (G) HCT116 cells was analyzed using dual luciferase reporter assay. Cells transfected with shCon or pcCon were used as controls. (**H**) Relative luciferase activity of p53short-Luc in YY2-silenced HCT116 cells was analyzed using dual luciferase reporter assay. Cells transfected with shCon were used as control. Relative luciferase activity was calculated as the ratio of the firefly and *Renilla* luciferase activities. Quantitative data were shown as relative to control, and were expressed as mean ± S.D. (*n* = 3). β-Actin was used for normalization in qPCR, and as a loading control in western blotting. NS: not significant; **P < 0.01; IP: immunoprecipitation; IB: immunoblotting.

The abovementioned results raised the question of how does YY2 regulate p53 activity. Protein degradation pathway is a crucial regulatory mechanism of p53 homeostasis [19, 20], and YY1 had been reported to bind and regulate p53 protein stability [8]. However, despite that YY2 positively regulates p53 protein accumulation (Figure 5A), immunoprecipitation results demonstrated that p53 could not be immunoprecipitated with YY2 (Figure 5B top panels), while it could be found in the immunoprecipitant of YY1 (Figure 5B bottom panels). These results indicated that YY2 might be involved in p53 regulation in its transcriptional level.

Indeed, the expression patterns of p53 was similar with YY2: its expression is downregulated in the breast cancer cell lines MCF-7 and MDA-MB-231 (Supplementary Figure 4A). Furthermore, we also revealed that YY2 silencing reduced the mRNA expression level of p53 in HCT116 cells, while YY2 overexpression upregulated it (Figure 5C). Thus, we next tried to analyze the promoter sequence of p53, and found a predicted YY2-specific binding site (AGAAGTGG) [21] at the –1,673 to –1,666 bp of the p53 promoter region (Figure 5D). To further confirm the role of this binding site in YY2 regulatory mechanism on p53 transcriptional activity, we constructed two kinds of p53-reporter vectors: p53-Luc with the –2,389 to +17 region of p53 promoter containing the predicted YY2 binding site; and p53short-Luc with the –1,629 to +17 region of p53 promoter lacking the predicted YY2 binding site (Figure 5E). We found that YY2-silencing grossly decreased the luciferase activity of p53-Luc reporter vector, while YY2 overexpression robustly induced it (Figure 5F and 5G, respectively). However, when the p53short-Luc reporter vector which lacks of YY2 binding site was used, YY2-silencing could not affect the luciferase activity (Figure 5H). Together, these results demonstrated that YY2 regulation on p53 occurs at its transcriptional level. Furthermore, the robust downregulation of p53 expression due to YY2-silencing could also be detected in breast cancer cell line MCF-7 and hepatocarcinoma cell line HepG2 (Supplementary Figure 4B, 4C), suggesting that this regulatory pathway could be found in various tumors.

**Figure 6:**
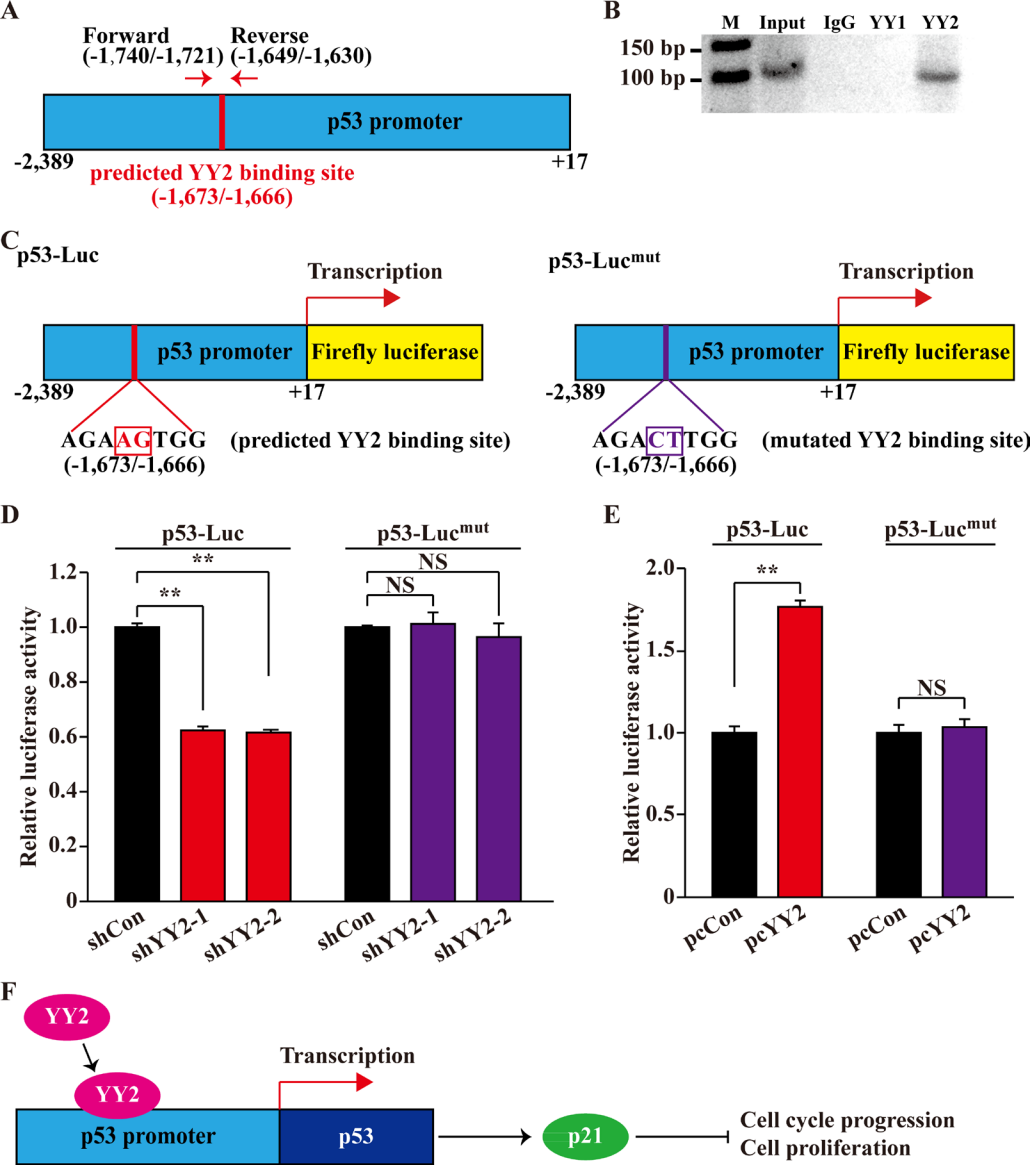
YY2 binds p53 promoter region (**A**) Schematic diagram of YY2 predicted binding site on p53 promoter region, and the location of primer set used for chromatin immunoprecipitation assay. (**B**) Bindings of YY1 and YY2 to p53 promoter region in HCT116 cells were examined using chromatin immunoprecipitation assay with anti-YY1 or anti-YY2 antibodies. PCR was performed using the primer set as described in (A). (**C**) Schematic diagrams of wild-type p53-reporter vector (p53-Luc, left) and YY2 binding site-mutated p53-reporter vector (p53-Luc^mut^, right). Mutated base pairs were indicated by squares. (**D**, **E**) Relative luciferase activities of p53-Luc and p53-Luc^mut^ in YY2-silenced (D) or YY2-overexpressed (E) HCT116 cells were analyzed using dual luciferase reporter assay. Cells transfected with shCon or pcCon were used as controls. Luciferase activity was calculated as the ratio of the firefly and *Renilla* luciferase activities. Quantitative data were shown as relative to control and expressed as mean ± S.D. (*n* = 3). NS: not significant; **P < 0.01. (**F**) Schematic diagram of YY2 regulation on the p53/p21 axis.

Next, to investigate whether or not YY1 and YY2 could bind to p53 promoter region at the predicted binding site as mentioned above, we performed ChIP assay by using anti-YY1 and anti-YY2 antibodies, and a primer set flanking the predicted binding site (Figure 6A). As shown in Figure 6B, we found that YY2, but not YY1, could bind to the -1,740 to -1,630 region of the p53 promoter. To further confirm whether the binding of YY2 on the predicted binding site affects its regulation on the p53 transcriptional level, we constructed a p53-Luc^mut^ reporter vector with two point mutations at the predicted binding site: the AGA*AG*TGG sequence in wild type p53 promoter was mutated into AGA*CT*TGG (Figure 6C). As shown in Figure 6D, YY2 silencing could substantially suppressed the luciferase activity of p53-Luc reporter vector, however, it did not affect the luciferase activity of p53-Luc^mut^ reporter. Concomitantly, YY2 overexpression could not induce the luciferase activity of this mutant reporter vector (Figure 6E). Together, these results showed that YY2 is a novel transcriptional regulator of p53 that binds to the –1,673 to –1,666 of the p53 promoter region.

## DISCUSSION

Unlike YY1, the expression patterns and biological functions of YY2 are poorly characterized. In this study, we showed that YY2 demonstrated an expression pattern opposite to YY1 in tumor and normal tissues. We identified YY2 as a tumor suppressor that positively regulates the transcriptional activity of tumor suppressor p53, and thus play a critical role in regulating conventional p53 pathway in a way contrary to YY1. Furthermore, we revealed that this regulatory function of YY2 could be found in various tumor cell lines, indicating that it is common in tumorigenesis.

A recent study reported that at developmental stages, YY2 expression shows dynamic changes in cerebellum and neocortex during gestation, neonatal and post-natal development; while YY1 expression remains unchanged [16]. In neuronal and glial cells, where YY1 is highly expressed, the expression level of YY2 is very low [15]. Furthermore, only YY1 could be detected in ovary follicles and sperm cells, while only YY2 could be detected in the granular layers of cerebellum [15]. Together, these facts strongly suggested that YY1 and YY2 different in their expression patterns, indicating that they are regulated distinctly and might exert different biological functions. Indeed, as described by Luo *et al*., YY2, which is located inside the Mbtps2 locus between the exon 5 and 6, was duplicated from YY1 mRNA through retroposition, and thus, did not duplicate YY1 regulatory regions for transcription [15].

Regarding the regulatory mechanism of YY2 expression, previous studies suggested two different possibilities: (1) YY2 and Mbtps2 showed similar spatial expression patterns in brain, ovary and testis, indicating that the two genes might be subject to similar transcriptional controls [15]; (2) other reports indicated that the two genes might also be regulated independently, as the expression of YY2 showed dynamic changes in neocortex and cerebellum during development, while that of Mbtps2 remained unchanged; and the expression of YY2, but not Mbtps2, could be regulated by DNA methylation [16, 22]. Our results showed that similar to that of YY2, the expression of Mbtps2 was downregulated in breast cancer cell lines (Supplementary Figure 5). However, further detailed analysis is needed to reveal whether or not YY2 and Mbtps2 share similar transcriptional control, or they are regulated separately by, for example, promoter methylation. Moreover, whether or not these regulatory mechanisms are responsible for the aberrant YY2 expression in tumor cells remains to be elucidated. Nevertheless, our results provide a new insight regarding the expression patterns of Mbtps2 and YY2, especially in tumor.

Despite that YY2 and YY1 are highly similar in their structures, our knowledge regarding the biological functions of YY2 is still very limited. Recent reports showed that YY2 might exert its own, specific functions. YY2 could adverse the transcriptional activator effect of YY1 on interleukin-4 (IL-4) by competing with YY1 for the same DNA binding sites on IL-4 promoter [23], and could antagonize the effect of YY1 on regulating the expression of beta interferon [24]. On the other hand, a very recent study described the function of YY2 in controlling embryonic stem cell self-renewal and differentiation [25]. However, to our knowledge, the role of YY2 on tumorigenesis remained unknown. Our study is the first one which showed that the decrease of YY2 might result in the suppression of tumor suppressor p53 and p21 expressions, and subsequently lead to the acceleration of cell cycle progression and cell proliferation. Despite that p53 is mutated in approximately half of all human malignancies, aberrant p53 expression due to the abnormal expression of its regulator could also be observed in many tumors with wild-type p53 [26]. Thus, our study unravel that the defect of p53 expression in tumor cells might be, at least partly, due to the lack of YY2 expression. Furthermore, as p53 is a fundamental, critical regulatory factor that also involved in many biological functions including apoptosis, DNA repair, senescence and embryogenesis, our results indicate the possibility that YY2 might also be involved in a broad range of biological aspects. Indeed, concomitant with the fact that YY2 positively regulates p53 expression, YY2 also exerted contrary effect with YY1 in apoptosis: YY2-silencing decreased the activity of caspase 3/7 (Supplementary Figure 6A–6B). Therefore, our results provide a new evidence that like YY1, a fine regulation of YY2 expression is also crucial for tumorigenesis. Furthermore, given that YY2 shares a high homology with YY1, and that YY2 could antagonize YY1 function in tumorigenesis, our study showed the importance for designing YY1-specific drugs, such that they specifically target the oncogenic YY1, but not YY2.

Our results identified a YY2 binding site in the promoter region of p53, and revealed that YY2 positively regulates p53 transcriptional activity, antagonizing the effect of YY1. Previous studies showed that YY2 could bind to some of the promoters containing YY1 binding sites but with lower DNA-binding affinity, possibly due to the phosphorylation of its amino acid residue 306 [13, 14, 22, 27, 28]. However, the YY2 binding site we identified here is predicted as a specific binding site of YY2, but not that of YY1 [21]. Furthermore, our results also showed that only YY2 could bind to this binding site. Thus, our results strongly suggest the presence of YY2 regulatory mechanism distinct from that of YY1.

Collectively, as described in the schematic diagram in Figure 6F, in this study we elucidated a novel, pivotal function of YY2 in regulating p53/p21 axis, which might lead to its specific role as a negative regulator of cell cycle progression and as a tumor suppressor. Our novel findings not only unraveled the importance of the poorly characterized YY2 and suggested that it might be a pivotal regulatory factor in more fundamental biological functions, but also give a new perspective on the regulation mechanism of conventional p53/p21 axis.

## MATERIALS AND METHODS

### Plasmids and constructs

Based on the results of applying our previously reported algorithm [29], we designed the RNA interference target sequences specific for YY1: shYY1-1 (5′-GCAAG AAGAGTTACCTCAG-3′) and shYY1-2 (5′-GGCAGA ATTTGCTAGAATG-3′); and for YY2: shYY2-1 (5′-GCA TCAACATCAACATCAA-3′) and shYY2-2 (5′-ACATCAA CATCAACCCAGA-3′). The shRNA expression vectors were constructed as described previously [30]. An shRNA expression vector containing a stretch of 7 thymines terminator sequences exactly downstream of the U6 promoter, namely shCon, was used as a control.

For p21 luciferase reporter vector, we cloned the -95 to +2,555 of the p21 promoter region into the *Bgl*II and *Hind*III sites of the pGL4.13 vector (Promega, Madison, WI). For p53 luciferase reporter vectors, we cloned the –2,389 to +17 (for p53-Luc) or the –1,629 to +17 (for p53short-Luc) of the p53 promoter region into the *Bgl*II and *Sph*I sites of the pGL4.13 vector. Human genome DNA was extracted from HCT116 cells using TIANamp Genomic DNA Kit (Tiangen Biotech, Beijing, China), and used as template for amplifying the promoter regions using the Takara Prime STAR Max DNA Polymerase (Takara Bio, Dalian, China). p21-luciferase vector lacking p53 binding site (p21(p53BSdel)-Luc) and p53-luciferase vector with mutated YY2 binding site (p53-Luc^mut^) were constructed base on the site-specific mutagenesis method [31].

For YY2 and p53 overexpression vectors (pcYY2 and pcp53, respectively), the coding regions of human YY2 and p53 were amplified as described above, and inserted into the *Bam*HI and *Not*I sites of pcDNA3.1(+) (Invitrogen Life Technologies, Carlsbad, CA). YY1 overexpression vector was constructed as previously described [30].

### Cell lines and cell culture

MCF-10A, MCF-7, MDA-MB-231 and HepG2 cells were maintained in Dulbecco's modified Eagle's medium (Gibco, Life Technologies, Grand Island, NY) with 10% fetal bovine serum (Biological Industries, Israel) and 1% penicillin-streptomycin. HCT116 and HCT116^p53-/-^ cells were kindly provided by Dr. Bert Vogelstein at The John Hopkins University Medicine School [32], and maintained in McCoy's 5A medium (Gibco) with 10% fetal bovine serum (Biological Industries) and 1% penicillin-streptomycin. All cells were cultured at 37^°^C in a humidified incubator with 5% CO2. Cells were transfected with indicated vectors using Lipofectamine 2000 (Invitrogen Life Technologies) according to the manufacturer's protocol. For gene-silencing experiments, 24 h after transfection, transfected cells were selected by using 1 μg/mL puromycin. All cell lines have been routinely tested for mycoplasma contamination using Mycoplasma Detection Kit-Quick Test (Biotool, Houston, TX). No cell line was found positive for mycoplasma.

### Clinical human breast carcinoma specimens

Human breast carcinoma fresh specimens were obtained from breast carcinoma patients undergoing surgery at Chongqing Cancer Institute (Chongqing, China). Patients did not receive chemotherapy, radiotherapy or other adjuvant therapies prior to the surgery. The specimens were snap-frozen in liquid nitrogen. Prior patients’ written informed consents were obtained, and the experiments were approved by the Institutional Research Ethics Committee of Chongqing Cancer Institute.

### Cell cycle analysis

Cells were transfected with indicated shRNA expression vectors and selected using puromycin selection as described above. Cells were then harvested and treated with propidium iodide (KeyGen Biotech, Jiangsu, China) for the detection of cell cycle according to the manufacturer's instructions prior to analysis using flow cytometry.

### RNA extraction and quantitative RT-PCR analysis

Total RNA was extracted with Trizol (Invitrogen Life Technologies) according to the manufacturer's instruction. Total RNA (1 μg) was reverse-transcribed into cDNA using the PrimeScript RT Reagent Kit with gDNA Eraser (Takara Bio), and quantitative RT-PCR was performed with SYBR Premix Ex Taq (Takara Bio) to assess mRNA expression levels. The sequences of the primers used for quantitative RT-PCR were shown in Supplementary Table 1. β-Actin was used to normalize sample amplifications.

### Western blotting

For cell culture experiments, cells were collected and lysed with RIPA lysis buffer with protease inhibitor and phosphatase inhibitor cocktail (complete cocktail; Roche Applied Science, Mannheim, Germany). For clinical specimens, the frozen specimens were homogenized with RIPA lysis buffer with protease inhibitor and phosphatase inhibitor cocktail (complete cocktail; Roche Applied Science) to obtain protein extracts. Equal amounts of the sample proteins were electrophoresed on sodium dodecyl sulfate polyacrylamide gel and transferred to a polyvinylidene fluoride (PVDF) membrane (Millipore, Billerica, MA). The antibodies used are shown in Supplementary Table 2. Immunoblotting with anti-β-Actin antibody was conducted to ensure equal protein loading. The signals were detected by using SuperSignal West Femto Maximum Sensitivity Substrate detection system (Thermo Scientific, Waltham, MA).

### Luciferase assay

Cells were co-transfected with indicated shRNA expression vectors or overexpression vectors, reporter vector bringing the firefly luciferase, and *Renilla* luciferase expression vector pRL-SV40 (Promega). Twenty-four hours later, luciferase reporter activities were analyzed by using Dual Luciferase Reporter Assay (Promega) as described previously [33].

### Immunoprecipitation assays

Cells were seeded in 10 cm dish (5 × 10^6^ cells/dish), and transfected with 16 μg overexpression vectors. Total protein samples were collected and lysed with RIPA lysis buffer with protease inhibitor and phosphatase inhibitor cocktail (complete cocktail; Roche Applied Science), and cleared by centrifugation at 12,000 rpm. The supernatants were incubated at 4°C for 2 h with protein A+G-beads (Beyotime Biotechnology, Shanghai, China) in the presence of indicated antibody or IgG as control. The immunoprecipitated proteins were then subjected to immunoblotting analysis as described in the western blotting section.

### Chromatin immunoprecipitation (ChIP) assay

ChIP analysis was performed using the ChIP-IT Express (Active Motif, Carlsbad, CA) according to the manufacturer's instructions. Briefly, to crosslink proteins to DNA, formaldehyde (final concentration 1%) was added to the culture medium of HCT116 cells overexpressing YY2. Cells were then collected, and the pellets were treated with Lysis Buffer prior to sonication to shear DNA into 0.2–1.0 kb fragments. After the cellular debris was removed, the chromatins were immunoprecipitated using protein G Magnetic Beads and anti-YY1 antibody, anti-YY2 antibody or normal rabbit IgG. Chromatin was then de-crosslinked for 15 min at 95°C prior to treatment with RNase A and proteinase K, and subjected to PCR analysis using PrimeSTAR Max (Takara Bio). The sequences of the primers used for PCR were: 5'-CCA AGC GCT GAA AGG AAG AT-3' and 5'-GAA GTG TGA GGT CGA TCT GT-3'.

### Immunohistochemistry and immunofluorescence

Clinical human breast carcinoma tissues were excised, freezed and sectioned into 10 μm slices using a cryostat. Tissue sections were incubated with a primary antibody for 1 h prior to staining with secondary antibodies conjugated with anti-rabbit Alexa Fluor 488 or anti-mouse Alexa Fluor 568 (Invitrogen Life Technologies). For Ki67 staining, cells transfected with indicated shRNA expression vectors and selected by using puromycin were re-seeded in a 3.5 mm cell culture dish. Cells were fixed with 4% paraformaldehide and permeabilized for 30 min with PBS containing 0.1% Triton X-100. After blocking with 2.5% bovine serum, the cells were incubated at room temperature for 1 h with primary antibody, followed by staining with secondary antibodies (anti-rabbit Alexa Fluor 488, Invitrogen Life Technologies). 4′,6-diamidino-2-phenylindole (DAPI, Beyotime, Guangzhou, China) were used to stain nuclei. Images were taken with Microsystems-TCS SP5 (Leica, Heidelberg, Germany). The antibodies used are shown in Supplementary Table 2.

### Hematoxylin and eosin staining

Clinical human breast carcinoma tissues were fixed with 4% paraformaldehyde for overnight prior to being embedded in paraffin and sectioned at 5 μm thickness using a cryostat. Sections were dewaxed using xylene and rehydrated before being stained with hematoxylin and eosin (Beyotime).

### Cell counting assay

Cells transfected with indicated shRNA expression vectors and selected by using puromycin were re-seeded in a 96-well cell culture dish 3 days after puromycin selection at a density of 4 × 10^3^ cells/well. Cell numbers were counted at indicated time points using colorimetric assays with 2-(2-methoxy-4-nitrophenyl)-3-(4-nitrophenyl)-5-(2,4-disulphophenyl)-2H-tetrazolium monosodium salt (Cell Counting Kit-8; Dojindo, Kumamoto, Japan) in accordance with the manufacturer's instructions.

### Caspase activity assay

Cells transfected with indicated shRNA expression vectors and selected by using puromycin were re-seeded in a 96-well cell culture dish. The caspase 3/7 activities were detected by using Caspase-Glo 3/7 Assay (Promega) according to the manufacturer's instructions.

### Statistical analysis

All values of the experimental results were presented as mean ± S.D. of triplicates. Statistical analysis was performed using Student's *t* test except for clinical samples, which was performed using one-way ANOVA. A value of *P < 0.05 was considered statistically significant.

## SUPPLEMENTARY MATERIALS FIGURES AND TABLES



## Abbreviations

YY family: Yin Yang family
YY1: Yin Yang 1
YY2: Yin Yang 2
p53: tumor suppressor p53
p21: cyclin dependent kinase inhibitor 1A (CDKN1A)
DNA: deoxyribonucleic acid
mRNA: messenger ribonucleic acid
Mbtps2: membrane bound transcription factor peptidase, site 2
shRNA: short hairpin ribonucleic acid
FACS: Fluorescence Activated Cell Sorter
CDK1: cyclin dependent kinase 1
IL-4: interleukin 4
RT-PCR: reverse-transcription polymerase chain reaction
ChIP assay: chromatin immunoprecipitation assay
