# Supplementary material for: Transcription factor Yin Yang 2 is a novel regulator of the p53/p21 axis
Source: Oncotarget. 2017 May 19;8(33):54694–707. doi: 10.18632/oncotarget.18005 (PMC5589614; doi:10.18632/oncotarget.18005)
Supplement: Supplementary file 1 [file oncotarget-08-54694-s001.pdf]

Transcription factor Yin Yang 2 is a novel regulator of the p53/p21 axis

SUPPLEMENTARY MATERIALS

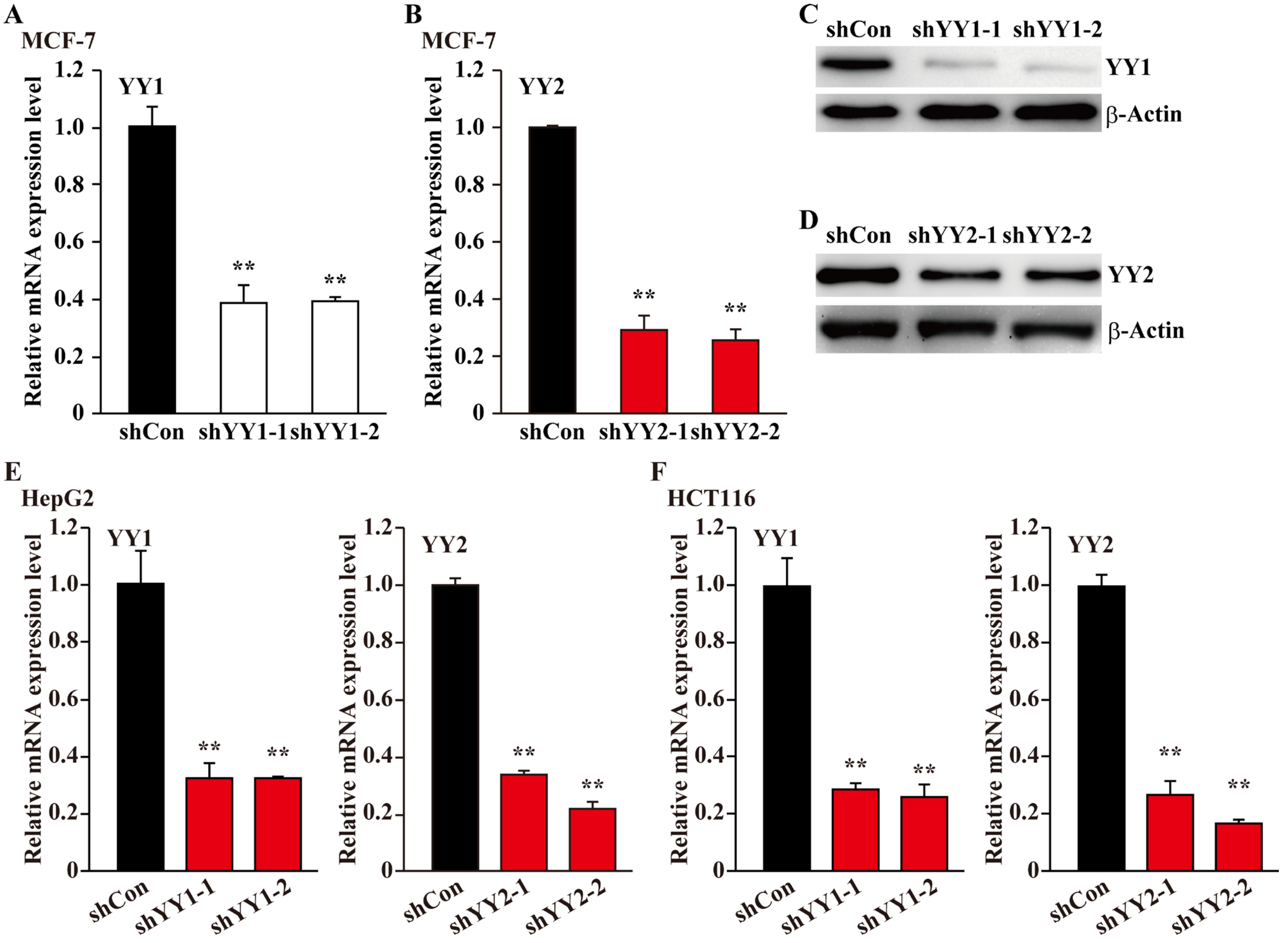

Supplementary Figure 1: The efficacies of YY1- and YY2-silencing using short hairpin RNA (shRNA) expression vectors. (**A**, **B**) YY1 (A) and YY2 (B) mRNA expression levels in the MCF-7 cells transfected with shYY1s or shYY2s were examined using quantitative RT-PCR (qPCR). (**C**, **D**) YY1 (C) and YY2 (D) protein expression levels in the MCF-7 cells transfected with shYY1s or shYY2s were examined using western blotting. (**E**, **F**) YY1 and YY2 mRNA expression levels in the HepG2 cells (E) and HCT116 cells (F) transfected with shYY1s or shYY2s were examined using qPCR. β-Actin was used for normalization in qPCR, and as a loading control in western blotting. Cells transfected with shCon were used as controls. Data were shown as relative to control and expressed as mean ± S.D. (n = 3). **$P < 0.01$.

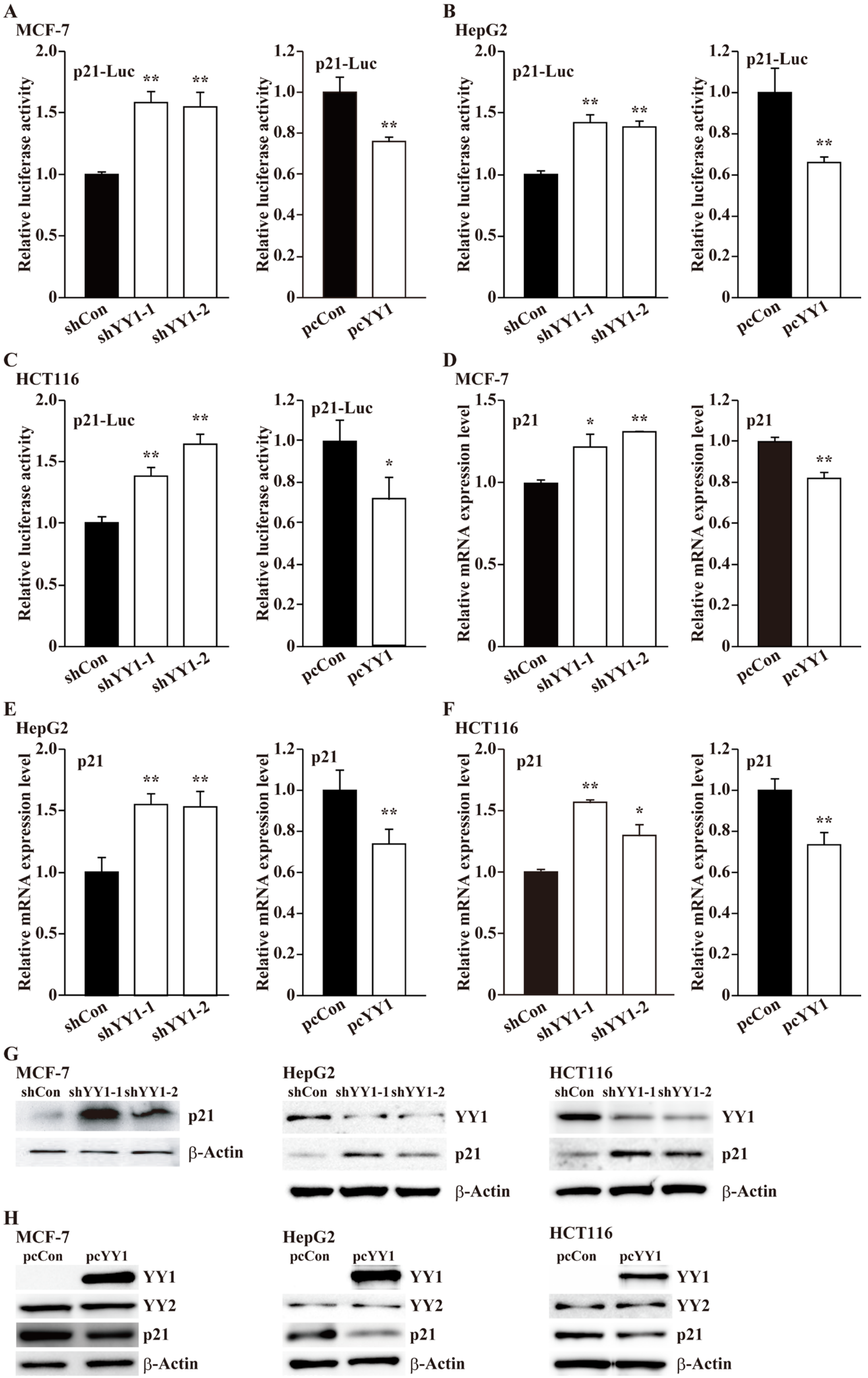

Supplementary Figure 2: YY1 suppresses p21 transcriptional activity. (**A–C**) The effect of YY1 silencing (left panels) and YY1 overexpression (right panels) on p21 reporter activity in MCF-7 (A), HepG2 (B) and HCT116 (C) cells was examined by using dual luciferase reporter assay. Cells transfected with shCon or pcCon were used as controls. Luciferase activity was calculated as the ratio of the firefly and *Renilla* luciferase activities. (**D–F**) p21 mRNA expression level in YY1-silenced (left panels) and YY1-overexpressed (right panels) MCF-7 (D), HepG2 (E) and HCT116 (F) cells was analyzed using quantitative RT-PCR. Cells transfected with shCon or pcCon were used as controls. β-Actin was used for normalization. (**G**, **H**) p21 protein expression level in YY1-silenced (G) and YY1-overexpressed (H) MCF-7, HepG2 and HCT116 cells was analyzed by using western blotting. β-Actin was used as a loading control. Quantitative data were shown as relative to control and expressed as mean ± S.D. ($n = 3$). pcCon: pcDNA3.1; * $P < 0.05$, ** $P < 0.01$.

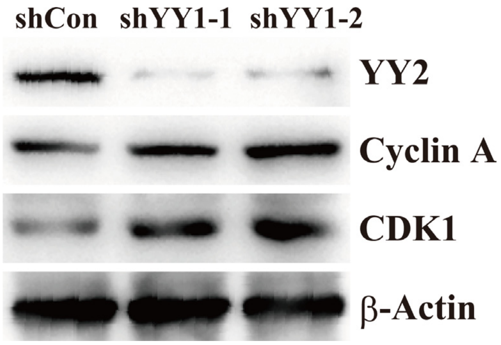

Supplementary Figure 3: YY2-silencing induces the accumulation of cell cycle related proteins. Protein expression levels of cyclin A and CDK1 in YY2-silenced HCT116 cells were examined using western blotting. β-Actin was used as a loading control.

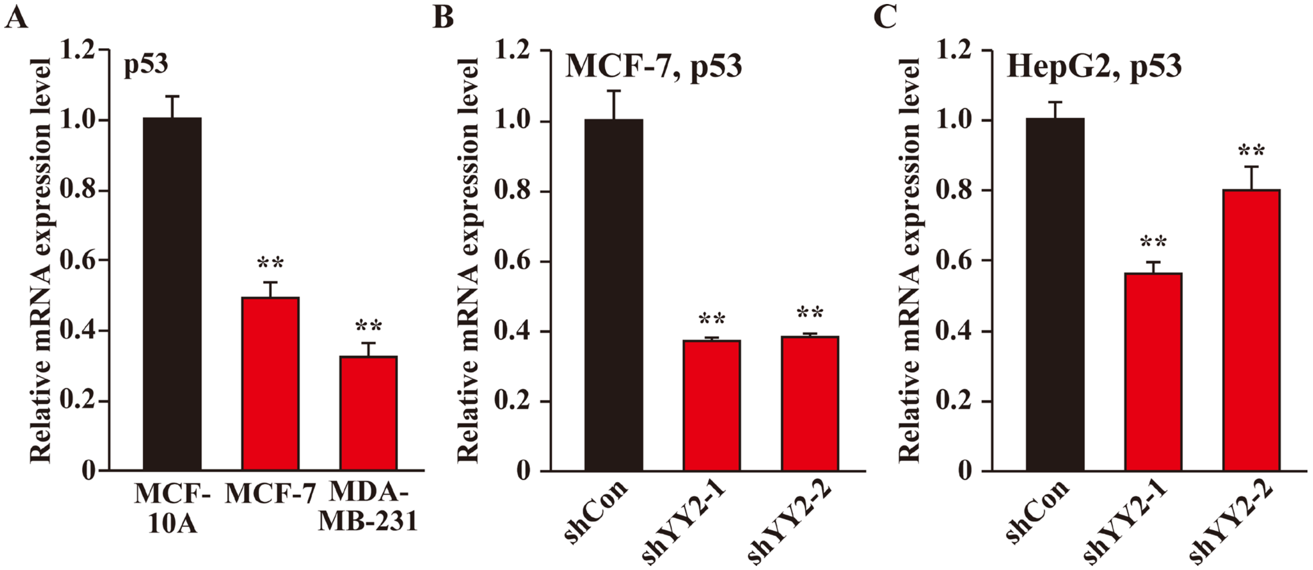

Supplementary Figure 4: YY2-silencing suppresses p53 expression level. (**A**) The mRNA expression level of p53 in MCF-10A, MCF-7 and MDA-MB-231 cells was analyzed using quantitative RT-PCR (qPCR). MCF-10A cells were used as control. (**B**, **C**) The mRNA expression level of p53 in YY2-silenced MCF-7 (B) and HepG2 (C) cells was analyzed using qPCR. Cells transfected with shCon were used as control. β-Actin was used for normalization. Data were shown as relative to control and expressed as mean ± S.D. (n = 3). $^{**}P < 0.01$.

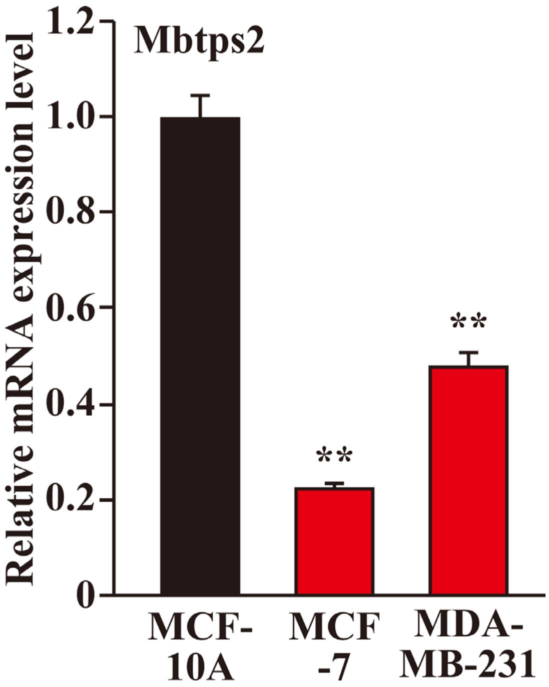

Supplementary Figure 5: Mbtps2 showed similar expression pattern with YY2. The mRNA expression level of Mbtps2 in MCF-10A, MCF-7 and MDA-MB-231 cells was analyzed using quantitative RT-PCR. MCF-10A cells were used as control. β-Actin was used for normalization. Data were shown as relative to control and expressed as mean ± S.D. (n = 3). **P < 0.01.

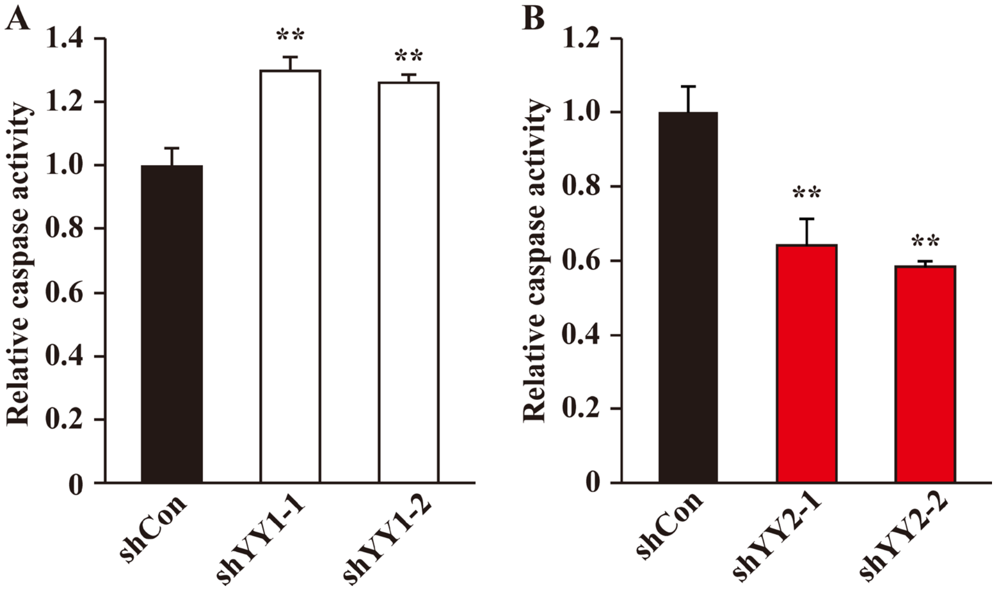

Supplementary Figure 6: YY2-silencing inhibits apoptosis. (**A**, **B**) The effects of YY1- (A) and YY2-silencing (B) on the activity of caspase 3/7 was measured. MCF-7 cells transfected with shCon were used as control. Data were shown as relative to control and expressed as mean ± S.D. (n = 3). **P < 0.01.

Supplementary Table 1: Primer pairs used for gene quantification by quantitative RT-PCR

Genes	Refseq No.	Forward primer sequence (5′–3′)	Reverse primer sequence (5′–3′)
YY1	NM_003403.4	GCGGAGCCCTCAGCCATGGCCTCG	CAGCGGCTGCAGAGCGATCATGG
YY2	NM_206923.3	GCAGTGGGTGAAGGCCAGGCTG	CGGTGTGGACCAGCTGGTGTCG
p21	NM_000389.4	TCACTGTCTTGTACCCTTGTGC	GGCGTTTGGAGTGGTAGAAA
p53	NM_000546.5	AGGCCTTGGAACTCAAGGAT	GGTAGACTGACCCTTTTTGGAC
Mbtps2	NM_015884.3	GGACATCCTCTGCATCTTCAC	GCTTAGAAAGTTAAAACGTGGGATA
β-Actin	NM_001101.3	CGAGCGCGGCTACAGCTT	TCCTTAATGTCACGCACGATTT

Supplementary Table 2: Antibodies used for western blotting, immunohistochemistrye and immunoprecipitation

Antibody	Product number	Maker	Experiment	Dilution
anti-YY1	#sc-1703	Santa Cruz Biotechnology	Western Blotting<br>Immunohistochemistry<br>Immunoprecipitation<br>Chromatin Immunopricipitation	1/5000<br>1/500<br>5 μg/ml cell lysate<br>30 μg/ml cell lysate
anti-YY2	Ab116507	Abcam	Western Blotting<br>Immunohistochemistry<br>Immunoprecipitation<br>Chromatin Immunopricipitation	1/1000<br>1/100<br>5 μg/ml cell lysate<br>30 μg/ml cell lysate
anti-CD31	#550389	BD Biosciences	Immunohistochemistry	1/100
anti-p21	sc-397	Santa Cruz Biotechnology	Western Blotting<br>Immunohistochemistry	1/50<br>1/50
anti-p53	sc-126	Santa Cruz Biotechnology	Western Blotting	1/1000
anti-Cyclin A	sc-751	Santa Cruz Biotechnology	Western Blotting	1/300
anti-CDK1	sc-54	Santa Cruz Biotechnology	Western Blotting	1/500
anti-β-Actin	#4967	Cell Signaling Technology	Western Blotting	1/10000
Goat Anti-Rabbit IgG	ZB2301	ZSGB-BIO	Western Blotting	1/10000
Goat Anti-Mouse IgG	ZB2305	ZSGB-BIO	Western Blotting	1/10000
Ipkine HRP Affinipure Goat Anti-Mouse IgG Light Chain	A25012	Abbkine	Immunoprecipitation	1/10000
Ipkine HRP Affinipure Mouse Anti-Rabbit IgG Light Chain	A25022	Abbkine	Immunoprecipitation	1/10000
Alexa Fluor 488 Donkey Anti-rabbit IgG	A21206	Invitrogen	Immunohistochemistry	1/500
Alexa Fluor 568 Goat Anti-mouse IgG	A11004	Invitrogen	Immunohistochemistry	1/100
Ki67	Ab15580	Abcam	Immunofluorescence	1/300
DAPI	C1006	Beyotime	Immunofluorescence	not diluted
